# Untethered soft actuators for soft standalone robotics

**DOI:** 10.1038/s41467-024-47639-0

**Published:** 2024-04-25

**Authors:** Yeongju Jung, Kangkyu Kwon, Jinwoo Lee, Seung Hwan Ko

**Affiliations:** 1 Applied Nano and Thermal Science Lab, Department of Mechanical Engineering, Seoul National University, 1 Gwanak-ro, Gwanak-gu, Seoul, 08826 South Korea; 2 Department of Mechanical, Robotics, and Energy Engineering, Dongguk University, 30 Pildong-ro 1-gil, Jung-gu, Seoul, 04620 South Korea; 3 Institute of Engineering Research / Institute of Advanced Machinery and Design (SNU-IAMD), Seoul National University, 1 Gwanak-ro, Gwanak-gu, Seoul, 08826 South Korea; 4 Interdisciplinary Program in Bioengineering, Seoul National University, 1 Gwanak-ro, Gwanak-gu, Seoul, 08826 Korea

**Keywords:** Mechanical engineering, Soft materials, Biomedical engineering

## Abstract

Soft actuators produce the mechanical force needed for the functional movements of soft robots, but they suffer from critical drawbacks since previously reported soft actuators often rely on electrical wires or pneumatic tubes for the power supply, which would limit the potential usage of soft robots in various practical applications. In this article, we review the new types of untethered soft actuators that represent breakthroughs and discuss the future perspective of soft actuators. We discuss the functional materials and innovative strategies that gave rise to untethered soft actuators and deliver our perspective on challenges and opportunities for future-generation soft actuators.

## Abstract (web summary)

Untethered soft robots offer numerous advantages in terms of mobility, versatility, and autonomy, making them increasingly valuable for a wide range of applications. Jung et al. review the new types of untethered soft actuators that represent breakthroughs and discuss the future perspective of soft actuators.

## Introduction

The recent advances in materials and innovative design architectures served to accelerate the development of soft actuators that provide the source of power for the locomotion of the robots. Unlike traditional robots that rely on rigid and bulky structures, soft robots are composed of materials that mimic biological tissues, such as elastomers or hydrogels, enabling them to mechanically deform like living organisms. Their flexible nature grants soft robots the ability to navigate complex environments, manipulate delicate objects, and interact safely with humans. Despite the revolutionary properties of soft robots and their potential to transform a variety of robotics industries, there exist critical technological challenges that soft robotics need to address. The technological limitations arise from the resultant weak actuation force to produce effective locomotion of robots, resulting in slow mobility and trivial force translation. However, a variety of technical and design breakthroughs have offered viable solutions to supply the power source and reinforce actuation force. Many of the recent soft robots resort to electrical wires and fluid tubes to supply an adequate power source for actuation^1,2^. Also, the recent strides in materials and mechanical design played a pivotal role in amplifying actuation force, exemplified by the incorporation of materials with variable stiffness and phase change materials^3,4^.

Yet, the most problematic hurdle, which even affects the overall performance and potential applications of soft robots, arises from the external wiring. Here, wires or wiring mean the external tether that connects the soft robot and the power source for the power supply: they do not refer to the electric wires inside the soft robot. A great number of the existing soft robots often rely on bulky pneumatic pumps or complex electrical wiring to supply the driving force for soft actuators via external power sources. Prior works on soft robots usually utilize the pump to create pressure or fluid flow, which drives the mechanical motion of the actuator, and electric motors can also generate mechanical motion with the electrical input. However, the incorporation of wires for the power source circumscribes the functionality and movement of soft robots despite the favorable characteristics of soft robots. For instance, when navigating through a tortuous path or reaching a site to which human beings do not access, the pneumatic tubes or wires exert a tension force on the robots and hinder their physical movements. In this regard, recent studies reported new types of soft actuators that do not accompany pneumatic pumps or electrical wirings to enhance the mobility of the soft robots and also to endow the robots with versatile functionalities.

For pneumatic actuators, the pneumatic pumps serve an essential role in generating a mechanical force by using compressed gas or moving the liquid for the rapid fluid pressure increase. Yet, the incorporation of the pneumatic pump into the soft robotics would impair the mobility and the core functionalities of the soft robots because the pumps are usually relatively bulky and heavy when compared to the soft robots themselves. To address this issue, several recent studies demonstrated pump-less pneumatic actuation by employing the phase change materials that generate the volume change as the materials switch between liquid and gaseous states, thus resulting in the inflation and deflation of actuators. Here, the pump-less pneumatic actuators can be defined as the soft actuators that do not use the actual pump but generate a pneumatic force by the phase change of material just as if utilizing the pneumatic pump. In other words, the pump-less pneumatic actuators just reproduce the end effect of the pump by a different working mechanism without using the actual pump. The absence of the pneumatic pump in the robotic design also eliminates the need for pneumatic tubes to infuse/extract air into/from the actuator, thereby making the design completely untethered.

Likewise, external stimuli can deliver a considerable amount of mechanical displacement and force needed to actuate the soft robots in an untethered manner: the external stimuli in this article include magnetic field, heat, electricity, light, and humidity. Hence, without physically connecting the electrical tethering to the soft actuators to provide the power source, the external stimuli can enable the soft actuator to produce mechanical displacement since the materials are designed to actuate as programmed. As opposed to the pneumatics-based soft actuators that require the onboard power source (such as a battery or self-powering energy harvesting devices) to supply power to induce pneumatic force, some of these actuators receive the power to induce mechanical displacement in a completely untethered fashion. For example, systematic manipulation of a magnetic field can control the movement of the magnet-driven soft actuator as intended without any type of wiring. Similarly, if the antennas are incorporated into the soft robotic system, electromagnetic waves can be utilized to provide power wirelessly to operate the soft actuators^5–8^, or it also enables the remote control of the actuators via wireless communication^9,10^ Therefore, external stimuli-driven soft actuators retain the potential to represent the breakthrough in the field of soft robotics although there exist considerable limitations to be resolved. In this light, it would be a highly valuable resource to introduce untethered soft actuators and discuss the future perspective of new types of soft robots. There are a considerable number of review articles on soft actuators and robotics^11–15^. However, no review paper has dealt with recent advances in untethered soft actuators for soft robotics that demonstrated meaningful outcomes within a few years. Recently, roboticists and researchers proposed an explosive number of soft actuators for soft robots based on innovative structural designs and functional materials that represent breakthroughs in the field of soft robotics. Furthermore, as the field of soft actuators is relatively new and drawing a substantial amount of interest in the related fields, there exists a demand for an article that systematically reviews the current trend and informs the opportunities to contribute to the field. In this regard, we believe that the timely and thorough review of the recent advances in untethered soft actuators will be informative for the general readers who wish to draw insights and gain potential perspectives in the field.

**Fig. 1:**
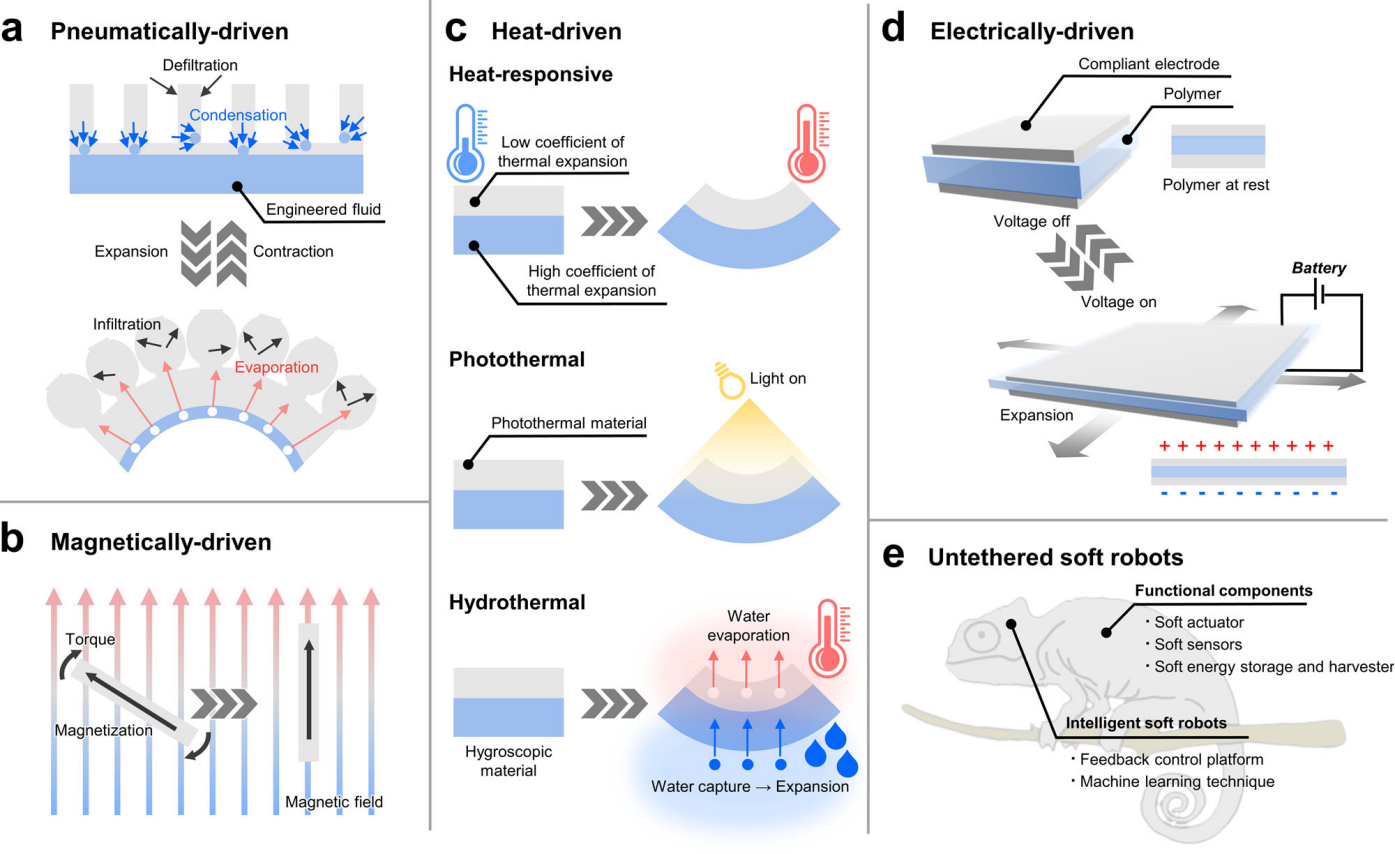
Graphical representation of different types of untethered soft actuators and their actuating mechanisms for soft robotics. **a** pneumatically-driven actuators. **b** magnetically-driven actuators. **c** heat-driven actuators. **d** electrically-driven actuators. **e** overview of the essential components for untethered soft robots. Pneumatically-driven actuators operate based on the volume change during phase change while magnetically-driven actuators utilize the interaction between magnetic fields and actuators made up of magnetic materials. Heat-driven actuators can be categorized into three types based on energy conversion: heat-responsive, photothermal, and hydrothermal. Electrically-driven actuators undergo mechanical deformation due to the charge polarization of dielectric materials in response to an applied electric field.

In this article, we introduce the representative works of the untethered soft actuators that serve as breakthroughs in soft robotics and further discuss the imminent challenges of the soft actuators to be addressed. Soft actuators can also be applied to rigid robots since the actuators reviewed in this paper operate in an untethered configuration. However, we intentionally circumscribed the scope and focused mainly on soft actuators for soft robots because the incorporation of soft actuators into the soft robot can make the entire robot soft and compliant. There exist specific applications where the soft robots exhibit comparative strengths over rigid robots such as navigating through the tortuous space^16,17^, exploring deep-sea at extremely high pressure^18^, or minimally invasive surgery^19^. Furthermore, to present these works systematically, the paper categorizes the soft actuators by four representative working mechanisms (1. pneumatically/hydraulically-driven, 2. magnetically-driven, 3. heat-driven, and 4. electrically-driven) and further examines each actuating mechanism in relation to the untethered soft robots as illustrated in Fig. 1. The paper examines the strengths and limitations of each actuating method and concludes with the future perspective of untethered soft actuators for soft robotics. Box 1 provides the general summary that addresses the strategies to provide the power source for actuation control of the soft robots. Additionally, Table 1 draws the overall comparison of each soft actuating method to highlight the strengths, weaknesses, and other key features such as response time and output force range. On the other hand, Table 2 captures key highlights of representative soft actuators that operate based on a variety of mechanisms and thus delivers a more specific comparison.

**Table 1:** Overall comparisons of soft actuators under different stimulation methods

Approach		Feature	Advantage	Limitations	Output Force Range	Response Time	Application
**Pneumatic/hydraulic**		Utilizes gas or liquid pressure for actuation	1. High force output 2. Fast response time 3. Versatile	1. Requires bulky pumps and fluid chambers 2. Limited untethered capability	Low to high	Fast	1. Soft grippers 2. Haptic devices 3. Bioinspired robots
**Magnetic**		Uses magnetic fields for controlled movement	1. Remote controllability 2. High spatial precision 3. Adaptability	1. Limited to magnetic field manipulation 2. Complex control over 3D motion	Low to medium	Slow to fast	1. Bioinspired robots 2. Micro/nanoscale actuators
**Heat**	**Heat-responsive**	Converts thermal energy into mechanical work	1. Energy efficient 2. High-force output 3. Design flexibility	1. Environmental Sensitivity 2. Slow response time 3. Safety concerns at high temps	Low to high	Slow to fast	1. Biomedical devices 2. Adaptive structures 3. Active hinges
	**Photothermal**	Utilizes light-induced heat for actuation	1. Rapid response 2. High precision 3. Non-invasive	1. Complex design for ultrafast actuation 2. Fiber diameter limitations 3. Miniaturization challenges	Low to high	Fast	1. Micro aerial vehicles 2. Ultrafast soft robotics 3. Programmable microfiber robots
	**Hydrothermal**	Responds to changes in humidity or moisture	1. Environmental sustainability 2. Biomimetic design	1. Limited sensitivity 2. Subtle humidity variation required 3. Uniformity challenges	Low to medium	Slow to moderate	1. Camouflage 2. Reconnaissance 3. Biomedical devices
**Electric**		Utilizes electrical input for actuation	1.Versatile actuation mechanisms 2.Mechanical flexibility 3.Swift response times	1. Complex control over 3D motion 2. May require high operating voltages 3. Environmental sensitivity in some cases	Low to high	Fast	1.Biomedical devices 2. Adaptive structures 3. Micro/nanoscale robots

**Table 2:** Representative soft actuators under different stimulation methods

	Material	Actuation methods	Performance	Response time	Ref
**Pneumatic**	Silicone elastomer (fluid chamber), Cu foam/paraffin (heatsink), Bi_2_Te_3_ (thermoelectric unit)	Liquid-vapor transition	16 N of inflating force within 175 s	0.47 mm/s and chamber width increase when inflating	^34^
	Silicone elastomer (fluid chamber), Bi_2_Te_3_ (thermoelectric unit)	Liquid-vapor transition	Speed of 0.515 BL/s	10 s to float to fully inflate, 14 s to fully deflate	^35^
	3D printed silicone-based materials	Pumping liquid	Pumping flow rate of ~ 521 ml/min	0.45 s	^48^
**Magnetic**	NdFeB, elastomer, magnetizer (SFY-2070)	Diaphragm segmented deformation	178.1 ml min^−1^ under −10 to 10 mT magnetic field with 10 Hz	178.1 ml /min	^50^
	Liquid metal coil, elastomeric substrates	Contract and expand by Lorentz forces	Speed 2.1 BL/s	(<0.97 mm/ms) for full stretch	^67^
	Magneto-responsive LCE with Fe_3_O_4_ NPS	Programmable shape deformation	80 % maximum contractile strain, magnetic controllability, multi-responsiveness, self-healing, remolding ability	60 s	^87^
	Magnetized NdFeB microparticles	Curling, Folding by pattern magnetization	Programmable magnetization profiles and functional modules	N/A	^93^
**Heat responsive**	LC ink (RM82, n-butylamine)	Rolling motion by thermal deformation	4D printing one time molding	Rolling speed 48 cm/min at 108 °C	^100^
	Silver nanowire and LCE	Shape deformation by joule heating	V > 5 V, bidirectional locomotion	0.5 mm/s (forward) and 0.72 mm/s (reverse)	^101^
	LCE bilayers	Bending and twisting	29 J/kg for 22 mg of LCE, lifted 450 times greater than the hinge mass	Rolling speed 12 cm in 95 s on the 200 °C hot plate	^102^
	Olefin copolymer, high density polyethylene, polymethyl methacrylate (PMMA)	Stretching and twisting by thermal expansion coefficient difference	Lift >650 times their own weight, withstand strains of >1000%	4 s for 50% linear contraption with $\Delta$T = 14 °C	^116^
**Photothermal**	Polyimide layer, low-density polyethylene (LDPE) layer, gold nanorods	Bending and twisting	Flight height of ~350 mm Flight time of ~8.9 s	N/A	^125^
	LCE microfibers, polydopamine coating layer	Elongation/contraction	Large actuation strain of ~ 60% High power density of ~ 400 W/kg	<0.2 s	^126^
	LCE with a reconfigurable constraint structure (fin-array-shaped)	Snapping motion	Moving speed of 2.5 m/s in launching and 0.22 m/s in jumping, long ejection distance of ~20 cm (35 mg ball), high jumping height of ~8 cm (40 times BL)	N/A	^127^
	LCE fiber, graphene filler	Elongation/contraction	Work capacity of 650 J/kg, power density of 293 W/kg	0.5 s at NIR intensity of ~2000 mW/cm^2^	^128^
**Hydrothermal**	Alginate-polyurethane hydrogel/yarn structure, poly(N-isopropylacrylamide)	Helical expansion/contraction	Maximum strain of ~16.2% (hot water of ~47 °C)	Expansion speed of 5.2 %/s, contraction speed of 3.1 %/s	^135^
	Pollen microgel	Bending	Steady-state curvature within ~80 s	N/A	^136^
	Hydrophobic cellulose acetate/hydrophilic polyacrylamide composite	Bending	Bending curvature of ~7.3 cm^−1,^ load capacity of up to 4.6 N/cm^2^	Response speed of ~10 degree/s	^137^
	Polypropylene, carbon black	Bending	Work up to 30.9 ×10−^2 ^J/kg, power up to 15.4 ×10−^2 ^W/kg	0.167 s	^138^
	Electrospun polyvinylpyrrolidone/poly(acrylic acid)/MIL-88A	Bending	Bending radius of 2.41 cm^−1^	0.084 cm^−1^ s^−1^	^163^
**Electric**	Dielectric elastomer material, power and control electronics	Flapping	Self-powered, actuated at a depth of 10,900 m, Swim freely at a depth of 3,224 m	Swimming speed 2.76 cm/s under 0.24 BL/s, 7 kV and 1 Hz	^18^
	Dielectric liquid, Silicone materials, tap water electrode	Extension with high voltage	~24 linear strain at 16 kV under a load of 300 g	~ 60 ms (water) and 52 ms (air) under a load of 300 g	^142^
	Conductive coiled yarn, polyester film	Switching	Origami multiplexed switches, logic gates, memory bits	1.5 ~ 1.7 s delay	^146^
	Shape memory polymer material, dielectric elastomer	Contract and expand	124 mm/s (backward), 112 mm/s (forward), 0.37 rad/s (angular velocity) at 5 − 8 kV and 0 ~ 50 Hz	N/A	^154^

Box 1 General overview that illustrates the solutions of power sources for actuation control methods in soft robots Distinguished by their flexibility, deformability, and adaptability, soft robots offer unique advantages over traditional rigid robots. However, soft actuators face significant challenges, particularly regarding power sources and external wiring because soft actuators require effective power sources for actuation to enable movement in soft robots. Recently, various options such as pneumatics, electricity, and magnetics have been explored, but the dependence on external wiring restricts mobility and functionality. Contemporary advancements have introduced soft actuators that eliminate the need for external wiring to enhance the capabilities of soft robots.

For instance, unlike the pneumatic actuators that traditionally rely on bulky pumps, innovations such as pneumatic actuation, based on phase change materials, circumvent this limitation, allowing for untethered operation. Additionally, external stimuli like magnetic fields, heat, and electricity can provide mechanical force, eliminating the need for physical connections and enabling greater mobility.

The article categorizes untethered soft actuators into four mechanisms: pneumatic/hydraulic-driven, magnetic-driven, heat-driven, and electric-driven, evaluating their strengths and limitations. Looking ahead, key challenges must be addressed for the widespread adoption of soft actuators, including reducing fabrication costs, ensuring manufacturing reproducibility, achieving precise force output, and enhancing material durability. Collaboration among researchers, industry, and the development of standardized practices will be crucial for advancing soft robotics and unlocking their potential across various applications.

## Diverse types of soft actuating methods and their operating mechanisms

### Pneumatically/hydraulically-driven soft actuators

**Fig. 2:**
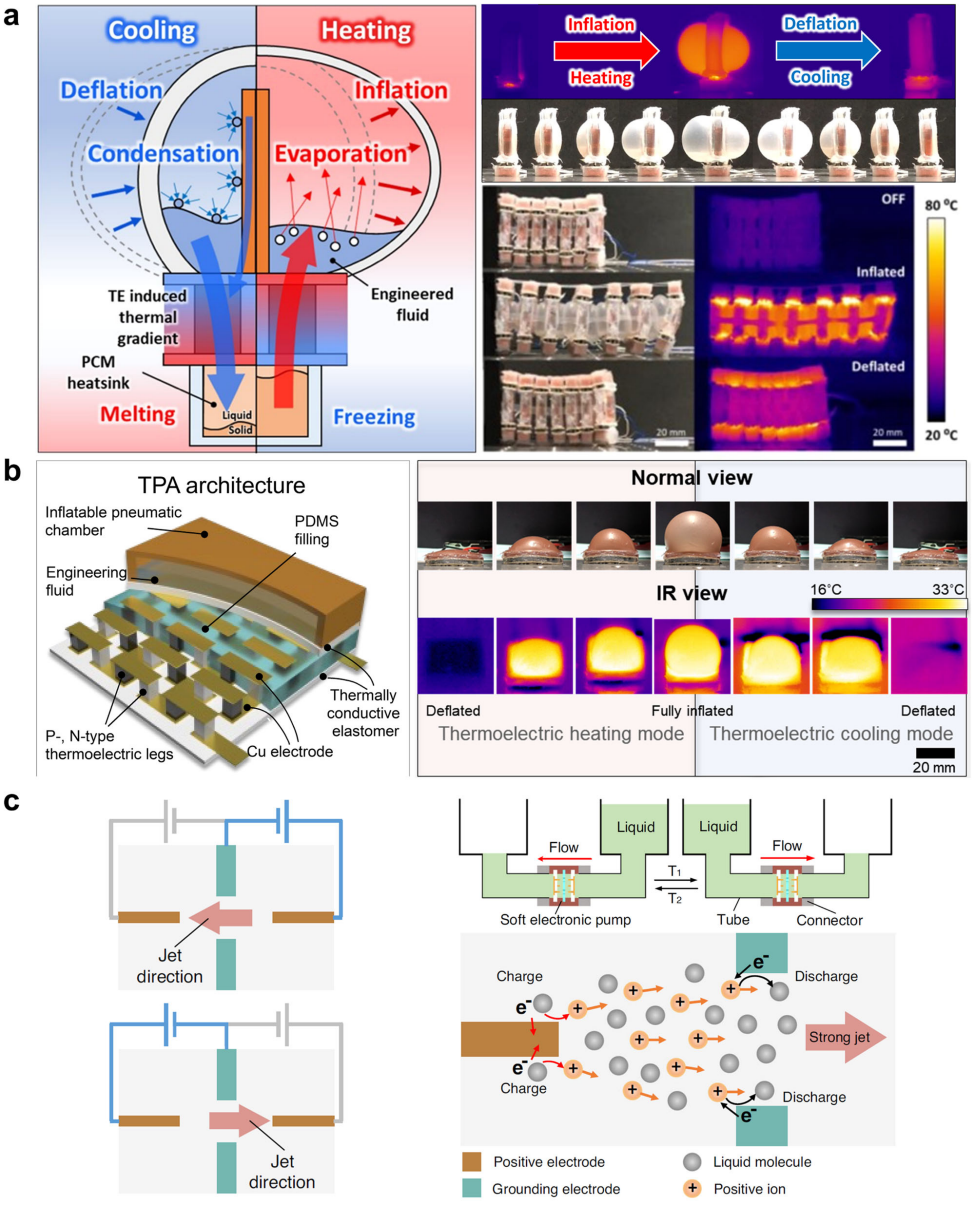
Untethered pneumatically -driven soft actuators. **a** Pumpless thermoelectric pneumatic actuator and soft earthworm robot. Reproduced with permission from ref. ^34^, copyright 2023, Elsevier BV. **b** Soft robotic fish based on the thermoelectric pneumatic actuator. Reproduced with permission under CC BY 4.0 license from ref. ^35^. **c** Soft electronic pump and robotic fish/vehicle based on the soft electronic pump. Reproduced with permission under CC BY 4.0 license from ref. ^48^.

Pneumatic and hydraulic actuators are capable of generating an enormous magnitude of mechanical force at a high response rate (~ 0.1 s) as they provide energy transmission through the mechanical properties of liquid and air^20^. For instance, soft pneumatic and hydraulic actuators are widely employed in practical applications such as soft grippers^21–26^, soft haptic devices^27^, and bioinspired robots^9,28–30^. However, pressurizing the pneumatic and hydraulic fluids requires pumps and fluid chambers that are usually too bulky and heavy to be integrated into the soft robot. Besides, utilizing the pumps and fluid chambers involves the pneumatic/hydraulic tubing to supply fluid to the actuator, undermining the core functionalities of the untethered soft robots. To address these issues, several studies developed soft pneumatic actuators that do not incorporate pumps but rather resorted to liquid-vapor transition to induce the dramatic volume change during the phase change^31–33^. The work in Fig. 2a utilizes a representative pump-less pneumatic actuator that operates based on the liquid-vapor transition to drive locomotion. The soft actuator in this study uses the flexible thermoelectric device to induce the inflation of the pneumatic chamber by liquid-vapor transition and, thus, to mimic the crawling movement of the earthworm^34^. The thermoelectric device offers desirable properties for pneumatic actuators since it can both cool and heat with a single device structure, suggesting that it can accelerate both liquid-to-vapor and vapor-to-liquid transitions. In addition, the thermoelectric device is lightweight, noise-free, and compact, making it a preferred candidate that worked based on the phase transition for the pump-less actuator. Exploiting the advantages of thermoelectricity, another study also developed a soft fish robot that can explore underwater space with the soft pump-less actuator, as depicted in Fig. 2b. The explosive volume change during the phase transition enables localized buoyancy control, which allows the soft fish robot to move forward and make a turn^35^. Both the pump-less soft actuators utilize untethered communications for piloting and onboard battery to provide the power source for thermoelectric devices, exhibiting a completely untethered configuration. However, despite the desirable features of the soft pump-less actuator based on the thermoelectric device, these actuators suffer from a relatively low response rate and thereby result in slow movements because phase change usually consumes a substantial amount of thermal energy. Other research groups also developed pneumatic actuators based on the liquid-vapor transition by actively heating (Joule heating) in order to control the buoyancy in the underwater environment^33,36^. Yet, these pneumatic actuators lack the cooling function to induce the vapor-to-liquid transition to lower the buoyant force by reducing the volume of soft actuators. Also, they showed a slow response rate to produce the buoyant force since the phase transition usually requires a substantial amount of energy. Several studies demonstrated pump-less soft actuators based on phase transition that was induced by the magnetic field. However, they do not show as high a response rate as conventional rigid robots^37–39^. Another way to achieve an untethered soft actuator based on pneumatics or hydraulics is to develop a soft and lightweight electronic pump that can be installed directly on the soft robot body. However, most of the recent studies on the soft pump involve pneumatic tubes or electrical wires^40–47^, except a few studies^48–50^. A representative study on an untethered soft electronic pump operates in the presence of a strong non-uniform electrical field that induces the movement of electrons and liquid molecules, and the liquid can move in both directions through the soft pump according to the electrical field. To design the soft actuator and robot completely untethered, the authors developed a lightweight miniaturized high-voltage converter that can transform the low-voltage input from the battery to high-voltage output for operating the soft pump. The soft miniature pump in this work can generate high hydraulic pressure and flow rate with a practical response rate (*t* ≈ 1 s), making it a highly potent candidate as the soft actuator for the soft integrated robot (Fig. 2c)^48^. Nevertheless, the volume of the fluid chamber can complicate the overall design of the soft robot because the greater the volume of the fluid chamber becomes, the bulkier and heavier the overall system of the soft robot gets. Yet, at the same time, the volume of the fluid chamber is directly proportional to the actuator capacity, so it requires meticulous design consideration to maximize its performance. Also, since the soft robots based on the soft electronic pumps do not support untethered communication for robot piloting, there still exists room for improvement.

In addition, although we classified the soft actuator research studies based on operating mechanisms, there are overlaps between specified categories because one arbitrary energy can be converted into another form to operate the actuation at the end. For instance, light energy can induce the liquid-vapor transition as an input energy such that the soft actuator eventually operates based on the pneumatics. A recent study by Shao et al. proposed the 4D printed, untethered soft actuator that undergoes volume expansion as a result of the liquid-vapor transition of methanol in the presence of the light input. Technically, when the light is illuminated on the actuator, the actuator absorbs light, and the absorbed light is converted to heat, which serves to cause the phase transition of methanol^51^. Such an actuation method based on light-driven phase transition does not require the onboard battery incorporation to provide power, making the entire structure lightweight and remarkably simple. Nonetheless, multiple energy conversion processes, for instance, from light to heat and then to the phase transition that leads to the volume change, indicate that there might be an energy loss during the energy conversion. The energy losses after a series of energy conversion processes can affect the overall efficiency of the actuation and slow down response time as a result. Thus, since there exists a clear trade-off between the structure complexity and energy efficiency, careful design considerations should be made according to the application of the soft robots.

To summarize, the recent advances in pump-less soft pneumatic and hydraulic actuators have shown great promise since they do not only require any pneumatic tubes connected to pumps and fluid chambers but also, at the same time, produce a sufficient amount of force to be used practically. However, the pump-less actuators rely on the bidirectional phase transition between liquid and gas that requires a considerable amount of thermal energy and thus often results in a poor response time. Alternatively, there have been efforts to develop a lightweight and compact pump for soft robotics such that the incorporation of the pump does not interfere with the overall design of the soft robots. Also, the light-driven pneumatic actuator presented a novel method to cause mechanical deformation based on a simple architecture that does not incorporate the onboard power source such as a battery. Even though the soft electronic pump and light-driven actuator offered immense potential as a future driving force to operate the untethered soft actuator, it has to be logically designed to enhance the overall performance of the soft robot.

### Magnetically-driven soft actuators

Magnet-driven untethered soft actuators, which are actuated by the influence of magnetic fields, draw inspiration from the way magnetic fields guide the behavior and movements of specific organisms. These actuators offer remarkable prospects for a wide range of applications. The advantages of using magnetic fields as an untethered external stimulus for actuating soft materials are distinct and multifaceted: remote controllability, high spatial precision, and adaptability across diverse environments. Magnetic fields have the unique ability to penetrate numerous materials, have freely generatable spatial gradients, and can be easily decoupled from other stimuli^52–55^. This makes them particularly versatile in shaping and controlling actuator behavior in response to external conditions. Moreover, a response of the material to changes in magnetic fields tends to be comparatively swift, rendering magnetic actuation an effective method for responsive and adaptive soft devices^56–58^. These advantages have shown great application potential for untethered soft actuators and robots such as bio-inspired designs^50,59–67^, magnetically-responsive materials^15,68–75^, micro/nanoscale actuators and robot^62,76–81^, and dynamic/reconfigurable structures^66,82–88^.

**Fig. 3:**
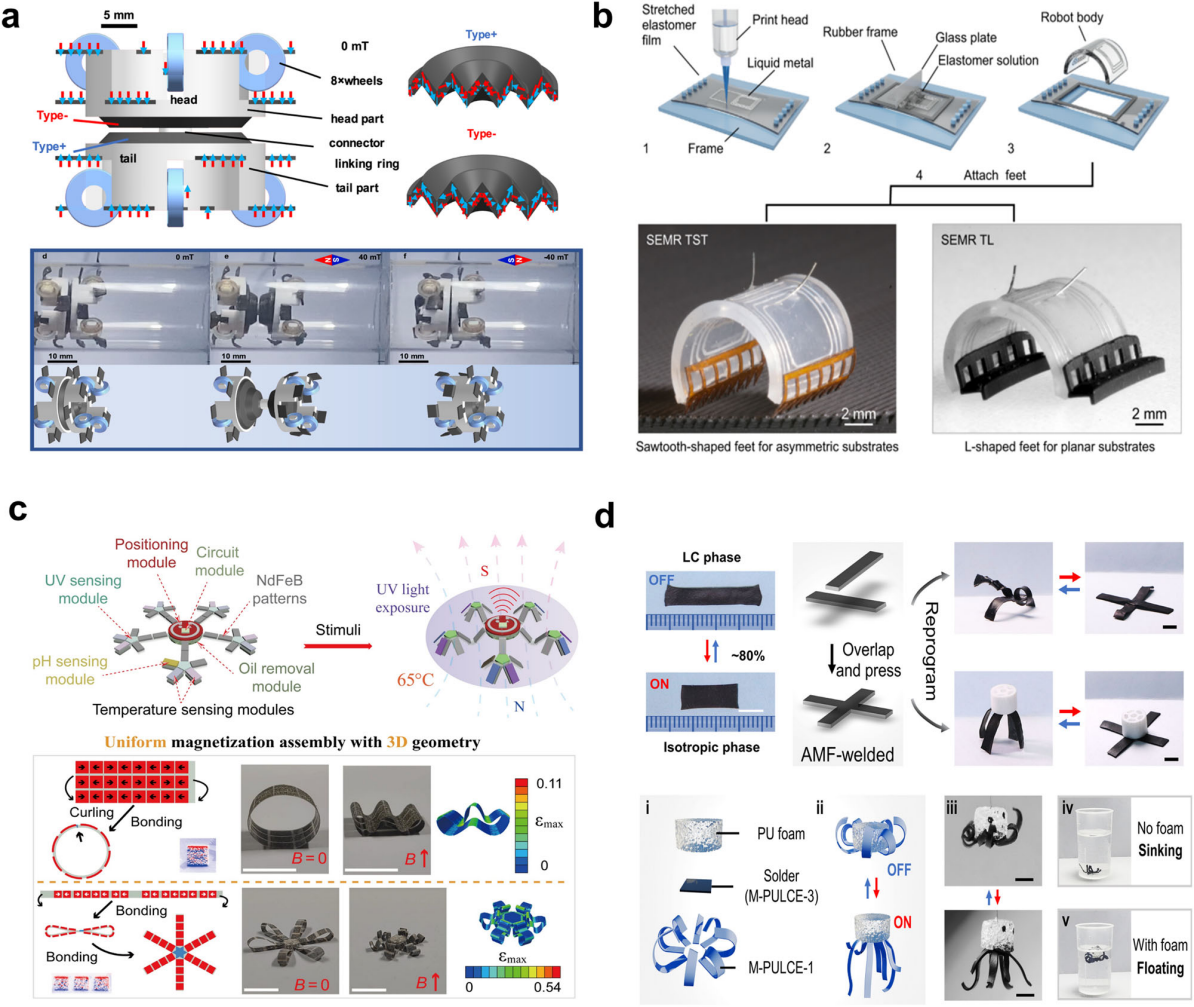
Untethered magnetically-driven soft actuators. **a** Bio-inspired magnetically-driven diaphragm and the soft robot based on the diaphragm actuator. Reproduced with permission under CC BY 4.0 license from ref. ^50^. **b** Small-scale soft electromagnetic robot made up of elastomeric bilayers. Reproduced with permission under CC BY 4.0 license from ref. ^67^. **c** Untethered small-scale magnetic soft robot with programmable magnetization. Reproduced with permission under CC BY-NC 4.0 license from ref. ^93^, copyright 2022, AAAS. **d** Untethered magnetic soft actuators based on liquid crystalline elastomers. Reproduced with permission under CC BY-NC 4.0 license from ref. ^87^, copyright 2022, AAAS.

Bio-inspired designs and dynamic, reconfigurable structures form the foundation for progress in the field of untethered magnetic soft actuators. Emulating the intricate movements of organisms, a group of researchers has created bio-inspired robots that mimic the locomotion of earthworms and squids (Fig. 3a). Their design utilizes a diaphragm formed from a single mold that is radially magnetized, which allows for significant two-directional and 3D deformations when exposed to a low homogeneous magnetic field^50^. Moreover, another work presented a type of miniature, bio-inspired soft electromagnetic robots built from curved bilayers of elastic material, propelled by the influence of Lorentz forces. The authors incorporated channels filled with printed liquid metal that transmit alternating currents, which is capable of versatile locomotion, including walking, running, swimming, jumping, steering, and cargo transportation at high speeds, as depicted in Fig. 3b^67^.

However, these advanced designs face substantial challenges since replicating bio-inspired movements solely is done by the modulation of magnetic fields. Unlike organisms that rely on a myriad of motor proteins and mechanical structures, magnetically driven actuators are limited to manipulating magnetic fields. Addressing these challenges, researchers focused on developing autonomous decision-making capabilities of bio-inspired magnetic soft robots, enabling them to intelligently respond to changes in magnetic fields, just as how biological organisms adapt their behavior based on sensory inputs^89–92^. A representative work in Fig. 3c utilized programmed magnetization patterns in soft robots, offering new possibilities for application related to multimodal robot locomotion with environmental sensing and detection^93^. In another research, the proposed fabrication strategy transforms 2D magnetic sheets into 3D soft magneto-active machines, enabling high-throughput production and various applications, including untethered biomedical robots, electronic robots, and mechanical encoders^93^. Still, inherent limitations in magnetic field control for complex three-dimensional movements present further difficulties, particularly over longer distances^55,94^. To address these challenges, researchers developed dynamic, reconfigurable, and magnetically responsive structures that can magnetically actuate soft materials with versatile contraction-derived motions and local magnetic control, as illustrated in Fig. 3d. Their work highlights the difficulties in achieving local and sequential magnetic control using multiple magnetization thresholds^87^.

Taken together, these exemplary works underline the recurring theme of challenges related to magnetic field manipulation. To overcome these hurdles and realize the full potential of magnetic soft robots, significant advancements in materials science and magnetic field control techniques are needed, thereby paving the way for the next generation of autonomous magnetic soft robots.

### Heat-driven soft actuators

Heat-driven actuators, an essential part of untethered soft robotics, possess unique characteristics and advantages since heat-driven actuators convert readily available thermal energy directly into mechanical work^11,95^. There exist several types of heat-driven soft actuators since energy in the other forms can be converted to heat that serves to induce the deformation of the actuators. Indeed, the most intuitive type would correspond to the actuators that respond to temperature change or heat itself (heat-responsive actuators). On the contrary, the actuators can also absorb light, which is converted into heat and serves to change the temperature of the specific actuator region, such that the actuators operate in the presence of light (photothermal actuators). Another type of actuator would be a hydrothermal actuator because the humidity or moisture is closely associated with temperature. Thus, this section will examine several types of representative heat-driven soft actuators in subsequent subsections.

### Heat-responsive soft actuators

**Fig. 4:**
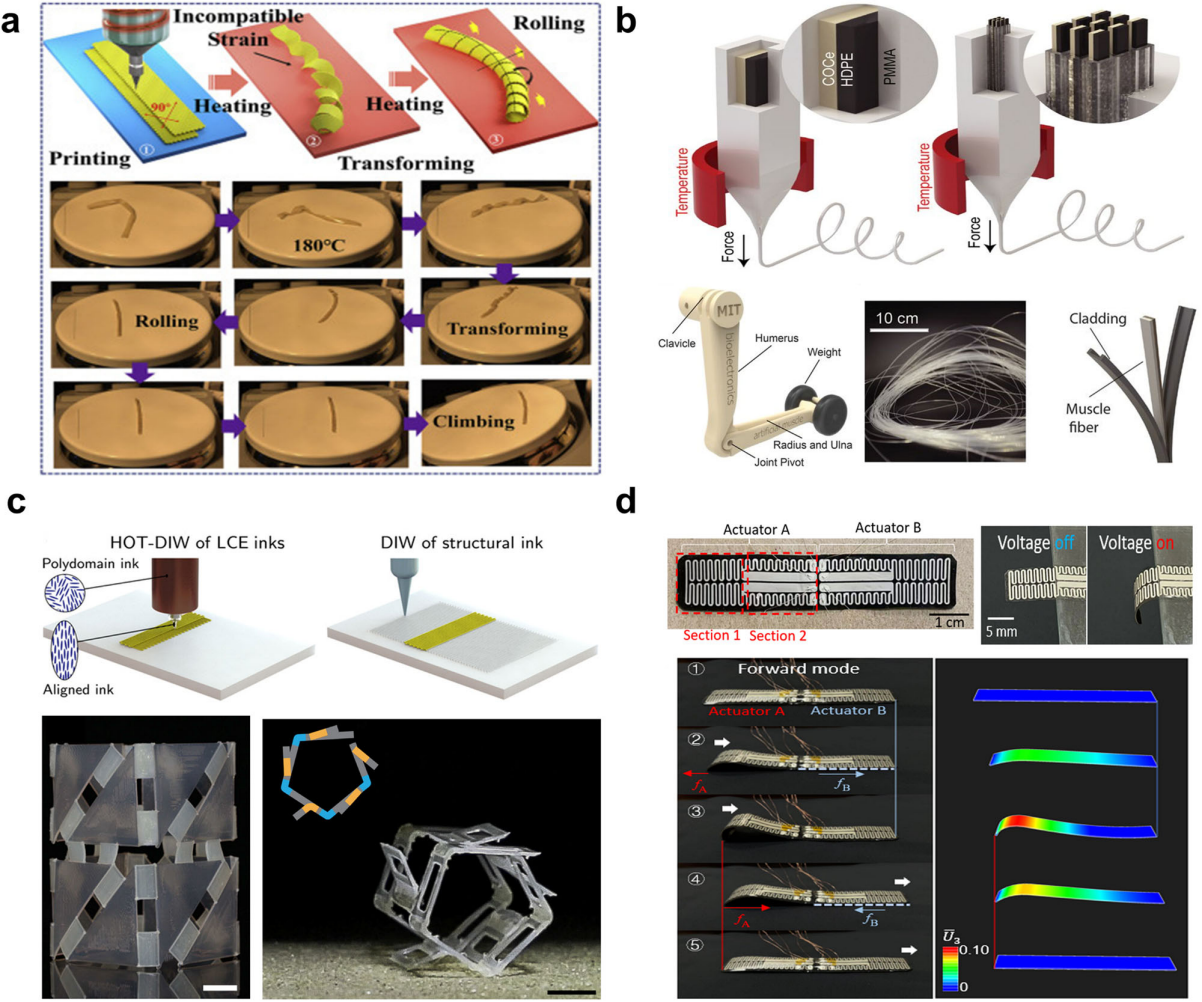
Untethered heat-responsive soft actuators. **a** 4D-printed untethered soft robot with tactile perception. Reproduced with permission from ref. ^100^, copyright 2021, Cell Press. **b** Artificial muscle soft actuator based on strain programmable fibers. Reproduced with permission from ref. ^116^, copyright 2019, The American Association for the Advancement of Science. **c** Untethered soft actuator with passive control of shape morphing. Reproduced with permission from ref. ^102^, copyright 2019. The American Association for the Advancement of Science. **d** Programmable heat-driven actuator and soft crawling robot. Reproduced with permission under CC BY 4.0 license from ref. ^101^.

Heat-responsive soft actuators undergo mechanical deformation in the presence of temperature change or heat exchange with the external environment. Heat-responsive actuators are primarily made of materials that are capable of undergoing considerable deformations to generate high force outputs when heated^96–98^, and these include materials such as liquid crystal elastomers (LCE)s^99–104^, hydrogel^105,106^, and shape-memory polymers (SMPs)^107–112^. One study demonstrated a 4D-printed untethered robot composed of LCE, which self-propelled when heated above 160 °C, illustrating the remarkable potential of heat-driven actuation^100^ (Fig. 4a). Twisted-and-coiled actuators (TCAs) hold promise for powering centimeter-scale soft robots due to their cost-effectiveness, high work density, and electrical operability, although their elevated temperature requirement may limit certain applications. Another research introduced an enhanced TCA fabrication method, enabling 48% free stroke capability and versatile motions in compact soft robots, with the potential for untethered operation using onboard electrical components^113^. Nonetheless, the elevated temperature threshold may limit its usage in certain applications, as it could lead to safety concerns or thermal damage to surrounding components. On another note, the relatively slow response time of heat-driven actuators can restrict their use in applications requiring rapid and high-frequency operation^114,115^. However, the relatively slow response time of heat-driven actuators can restrict their use in applications requiring rapid and high-frequency operation^114,115^. A notable work addressed this limitation by creating strain-programmable artificial muscles that demonstrated thermal and optical controllability, resilience across numerous deformation cycles, and a lifting capacity of >650 times their weight, as depicted in Fig. 4b^116^. While this work represents a significant advancement, improving the actuation speed without compromising the force output serves as an ongoing challenge in this field. Also, heat-responsive actuators often employ versatile materials that can be molded into diverse shapes and configurations to provide design flexibility and possible cost reductions. A representative study demonstrated the fabrication of active hinges using LCE bilayers and achieved significant reversible bending responses to thermal stimuli, resulting in self-assembling and self-propelling “rollbots“^102^ (Fig. 4c). Yet challenges persist in ensuring consistent response times and careful synchronization when employing multiple active hinges in a single system. Additionally, thermal-responsive poly(isopropylacrylamide-co-2-(dimethylamino)ethyl methacrylate)/alginate hydrogels have been developed, showcasing the adaptability of heat-responsive materials for intricate shape-changing behaviors and versatile application^106^. Researchers also explored new possibilities by combining electrical and thermal inputs to achieve complex motions with the ionic polymer-metal composite actuator, representing a significant breakthrough in controlling twisting and bending deformations^117^.

The sensitivity of heat-responsive actuators to environmental temperature also remains a notable challenge because unintentional actuation can occur due to ambient temperature fluctuations that might lead to unintended movements and reduced accuracy^118,119^. In an attempt to address this issue, a group of researchers used a caterpillar-inspired robot design and introduced a patterned soft heater to an LCE-based thermal bimorph actuator (Fig. 4d). This design utilized programmable heating to achieve different temperature and curvature distributions, enabling bidirectional locomotion and better control^101^. Another example of a bioinspired robot design using thermally responsive hydrogels like poly(N-isopropylacrylamide) (PNIPAM) was demonstrated, enabling complex shape changes, including an artificial flower that blossoms and reverses with temperature shifts^105^. Despite the advancements, the fine-tuning of temperature control and heat distribution still presents a significant hurdle, especially when scaling to larger systems.

In summary, heat-responsive actuators offer several advantages, including manufacturing simplicity and high-force output, making them attractive for various soft robotics applications. These benefits stem from their ability to convert thermal energy into mechanical work, which can lead to reduced energy consumption, passive actuation capabilities, and sustainability in certain scenarios. However, it is crucial to acknowledge the challenges these actuators face, such as environmental sensitivity, response time limitations, and safety concerns in high-temperature operations. For example, their performance may be affected by ambient temperature fluctuations, potentially resulting in unintended movements or reduced precision. Achieving optimal energy efficiency often requires precise temperature control, which can be challenging, especially when scaling up or operating in dynamic environments. Researchers are actively working to enhance the overall performance of heat-responsive actuators, aiming to maximize their benefits while mitigating limitations.

### Photothermally-driven soft actuators

Natural species have evolved to thrive in specific environmental lighting conditions, often evolving their mobility responses to light, as seen in sun-tracking plants and photoreceptive muscle fibers. Drawing inspiration from these biological mechanisms, numerous studies have extensively employed light as a untethered external photothermal stimulus source to drive the motion of soft actuators in the past decades^120–124^. Light offers clear advantages as an external stimulus source for soft actuators due to its ability to facilitate the motion control of soft actuators in rapid response, high precision, and non-invasive manners, which is achieved by modulating various light parameters, including wavelength, intensity, and polarization to leverage diverse operating mechanisms.

**Fig. 5:**
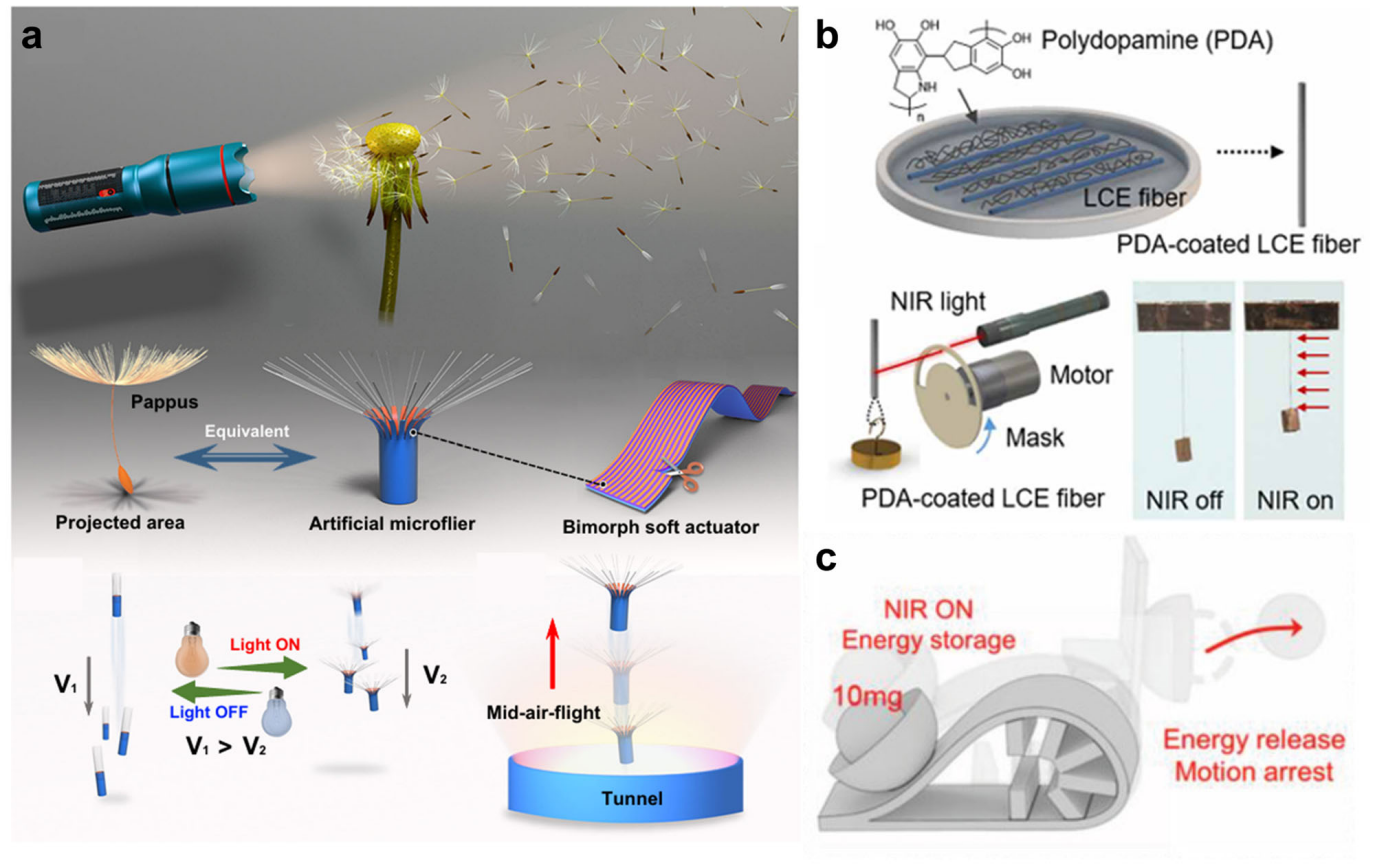
Untethered photothermally driven soft actuator. **a** Artificial microflier inspired by dandelions and their components. Reproduced with permission under CC BY 4.0 licence from ref. ^125^. **b** Laser-driven soft actuator based on electrospun LCE microfiber coated with PDA. Reproduced with permission from ref. ^126^, copyright 2019, The American Association for the Advancement of Science. **c** Light-initiated actuation of an LCE catapult with tunable and programmable snapping dynamics. Reproduced with permission from ref. ^127^, copyright 2023, Wiley.

An illustrative example of harnessing the photothermal effect can be observed in Fig. 5a, where a dandelion-inspired light-driven actuator was developed. This actuator employed gold nanorods to achieve selective optical absorption and high photothermal conversion efficiency, aiming to mimic the motion of insect-scale micro aerial vehicles^125^. The dandelion-inspired actuator, characterized by its tubular shape, addresses the issue of randomly-flighting motion typically associated with conventional artificial dandelion devices. Consequently, it allowed for controlled rotation in both clockwise and counterclockwise directions. Additionally, liquid crystal elastomers (LCEs) have also been widely utilized for photosensitive actuators. The micro-scale fiber actuator was developed through the utilization of an electrospinning technique applied to LCEs (Fig. 5b), exhibiting high actuation strain (~ 60%) with a rapid response time (<0.2 s)^126^. This innovative approach effectively addressed an issue associated with macro-scale LCE fibers (> 0.3 mm), characterized by slow actuation response. The results presented exceptional actuating performance comparable with that of human muscle fibers.

Moreover, to enhance the performance of a photothermally-driven soft actuator, the interesting work is presented in Fig. 5c^127^. Inspired by the snapping motions observed in various living organisms, the researchers utilized a reconfigurable structural constraint into a photosensitive LCE actuator that mimics the snapping motions observed in various living organisms. The novel approach resulted in an actuator with an exceptional rapid motion (up to 2.5 m s^−1^), achieved by effectively converting elastic energy to kinetic energy, implying that the incorporation of reconfigurable structure into soft actuating systems holds promise for the development of ultrafast soft robotics. Furthermore, to address the challenge of soft material with inherently weak mechanical properties, a group of researchers introduced nanofillers into polymer matrices. This approach was inspired by the human-muscle fibrous system^128^. Benefitting from the exceptional thermal and mechanical properties of graphene fillers, the resulting artificial muscle fibers displayed not only rapid actuation upon exposure to near-infrared (NIR) irradiation but also remarkable power density (293 W kg^−1^), which is about 6 times higher than that of human muscle. Notably, the reversible filler percolation network configuration effectively mitigates the inherent mechanical weakening of fiber-based soft actuators in the contracted state.

In summary, many researchers have focused on the diverse photothermally-driven soft actuators. However, there remains a need for future studies to address critical challenges associated with conventional approaches. For instance, for the microscale photothermally-driven actuator such as the microflier, a crucial consideration should be the miniaturization of electronic components to enable seamless integration into small-size actuators, thereby enhancing programmable controllability. Moreover, to achieve the rapid response for the fiber-based photothermal actuator, an effort should focus on reducing fiber diameter (<10 μm) because the larger fiber diameter results in a slow thermo-actuation response, as aforementioned. Besides, as the diameters of LCE fibers have been reported to greatly deviate (10 ~ 100 μm), it is of great importance to achieve high uniformity of fiber diameters for practical applications such as microfiber robots.

### Hydrothermally-driven soft actuators

In nature, humidity plays a significant role in influencing the behavior of living organisms through swelling or deswelling induced by water^129^. Moreover, it is worth noting that humidity and moisture levels are greatly affected by temperature variations. In this section, we demonstrate a soft actuator that responds not only to changes in humidity but also to solvent evaporation triggered by thermal stimuli. Among the various natural objects, pine cones are one of the representatives that exhibit deformation in response to different humidity conditions^130,131^. This hygroscopic behavior in pine cones is intricately linked to their ability to open or close in response to environmental humidity variations, driven by changes in the moisture content within the cones. This behavior originates from the substantial divergence in hygroscopic expansion coefficient among different tissues within the pine cone. Based on the simple mechanism, pine cone-inspired soft actuators have been widely reported in recent years^132,133^.

**Fig. 6:**
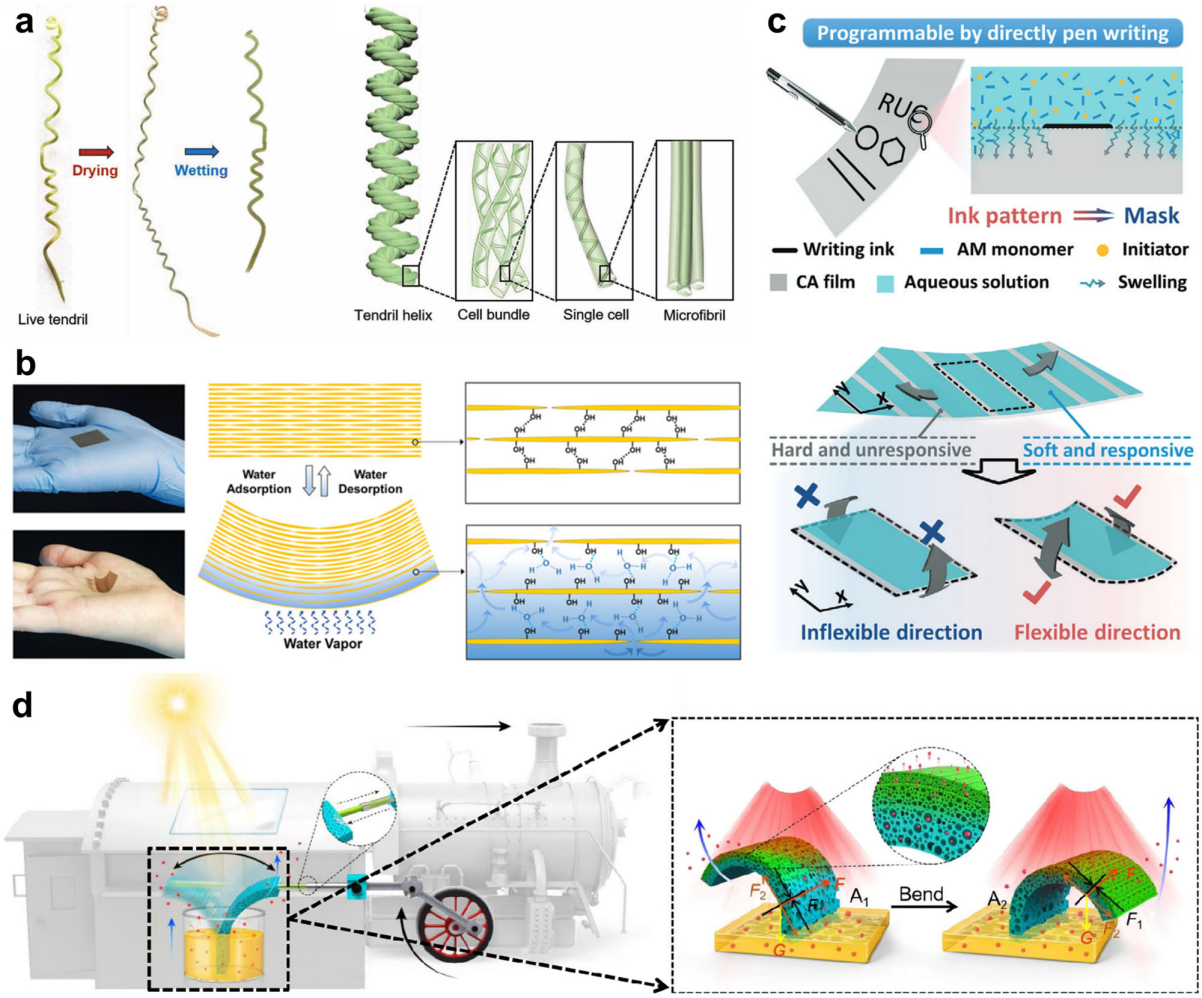
Untethered hydrothermally driven soft actuator. **a** Plant-inspired soft artificial muscles with tropism mechanism. Actual photograph of a live plant tendril (left) and graphical representation of plant-like actuator with macro-/micro components. Reproduced with permission from ref. ^135^, copyright 2023, Wiley. **b** Natural pollen paper-based humidity-driven soft actuator. Reproduced under CC BY-NC 4.0 license from ref. ^136^, copyright 2020, NAS. **c** Programmable actuation by direct pen writing. Reproduced with permission from ref. ^137^, copyright 2023, Wiley. **d** Light motor based on hydrothermal solvent evaporation. Reproduced with permission under CC BY 4.0 license from ref. ^138^.

Nevertheless, there exists an urgent need to further explore the intricate bending mechanisms of the pine cones, given the relative lack of research into the remarkably slow deformation process observed in pine cones as compared to other hygroscopic plants. In this regard, a group of researchers revealed that the ultra-slowly hygroscopic deformation of the pine cones is mainly attributed to the distinct vascular bundle structures characterized by spring/square microtube configurations^134^. By further exploring the hygroscopic behavior, which operates at an ultra-low speed and is primarily governed by the unique heterostructures, the researchers developed soft actuators. These soft actuators, integrated with the heterostructure through material design, facilitate imperceptible yet controllable motions, thereby offering valuable insights into the various fields where imperceptibility is crucial, including camouflage and reconnaissance. Moreover, Aziz et al. developed a plant-like artificial muscle at the micro level by imitating the helical plants in nature (Fig. 6a)^135^. The hierarchically chiral microstructures enable plant-like actuators to deform with a large stroke and rapid velocities, akin to natural plants that exhibit hydrotropism or thermotropism through hydrothermal volume expansion and contraction process induced by changes in humidity or heat.

As green electronics have extensively attracted significant attention in a variety of fields, environmentally sustainable actuators have been extensively explored. Researchers have developed eco-friendly paper-based actuators derived from naturally abundant pollen grains (Fig. 6b)^136^. The actuator exhibited asymmetric microstructure on the top and bottom surfaces induced by water evaporation during the fabrication process. This asymmetry resulted in distinct deformation degrees of top and bottom surfaces, enabling locomotion under different humidity conditions. The performance of the paper-based actuator can be fine-tuned by controlling the paper thickness and the fabrication process. Furthermore, Yang et al. developed a humidity-sensitive synthetic earthworm as a degradable actuator capable of emulating the behavior of natural earthworms. The unique ability to function in soil is facilitated by the capacity of water to penetrate through the soil^137^. Especially, the employment of pen-writing patterning technology into a bilayer film composed of hydrophobic/philic layers results in programmable actuation with more precise control in the desired ways, thereby enabling the actuator to operate as intelligent soft robots to mimic the behavior of natural worms (Fig. 6c).

The direct conversion of light energy into mechanical energy has attracted significant attention due to small efficiency losses, particularly when compared to conventional energy conversion methods such as photovoltaics, which involve multiple energy conversion steps and may result in energy losses. The research presented in Fig. 6d introduces a light-driven oscillator that operates based on a porous structure designed for rapid adsorption and desorption^138^. The solar engine-like oscillator exhibited a maximum power of (15.4 × 10^−2 ^W kg^−1^) and can ceaselessly operate under solvent supply. Thus, the oscillator holds significant promise for practical applications such as use in universe exploration and rescue operations in challenging, remote areas that are difficult for human beings. Its capacity to respond to diffuse light sources such as sunlight distinguishes it from the previous oscillators, which were primarily activated by tightly focused incident light, typically generated by laser beams.

Recently, the exploration of natural organisms as bionic models for hydrothermally-driven soft actuators has garnered considerable attention^139,140^. Likewise, hydrothermally-driven untethered soft actuators, which can operate under non-invasive conditions unlike other types of actuators driven by high electrical input, high temperature, and UV light, have attracted significant interest from a variety of researchers, thereby extensively employable in biomedical applications^141^. Nevertheless, for practical, real-world applications, there is a pressing requirement to develop highly sensitive hydrothermal-based actuators. These actuators must reliably function even within a subtle variation in environmental humidity, especially based on a deeper understanding of the operational mechanisms, not just shape-mimicking actuators.

### Electrically-driven soft actuators for soft robots

**Fig. 7:**
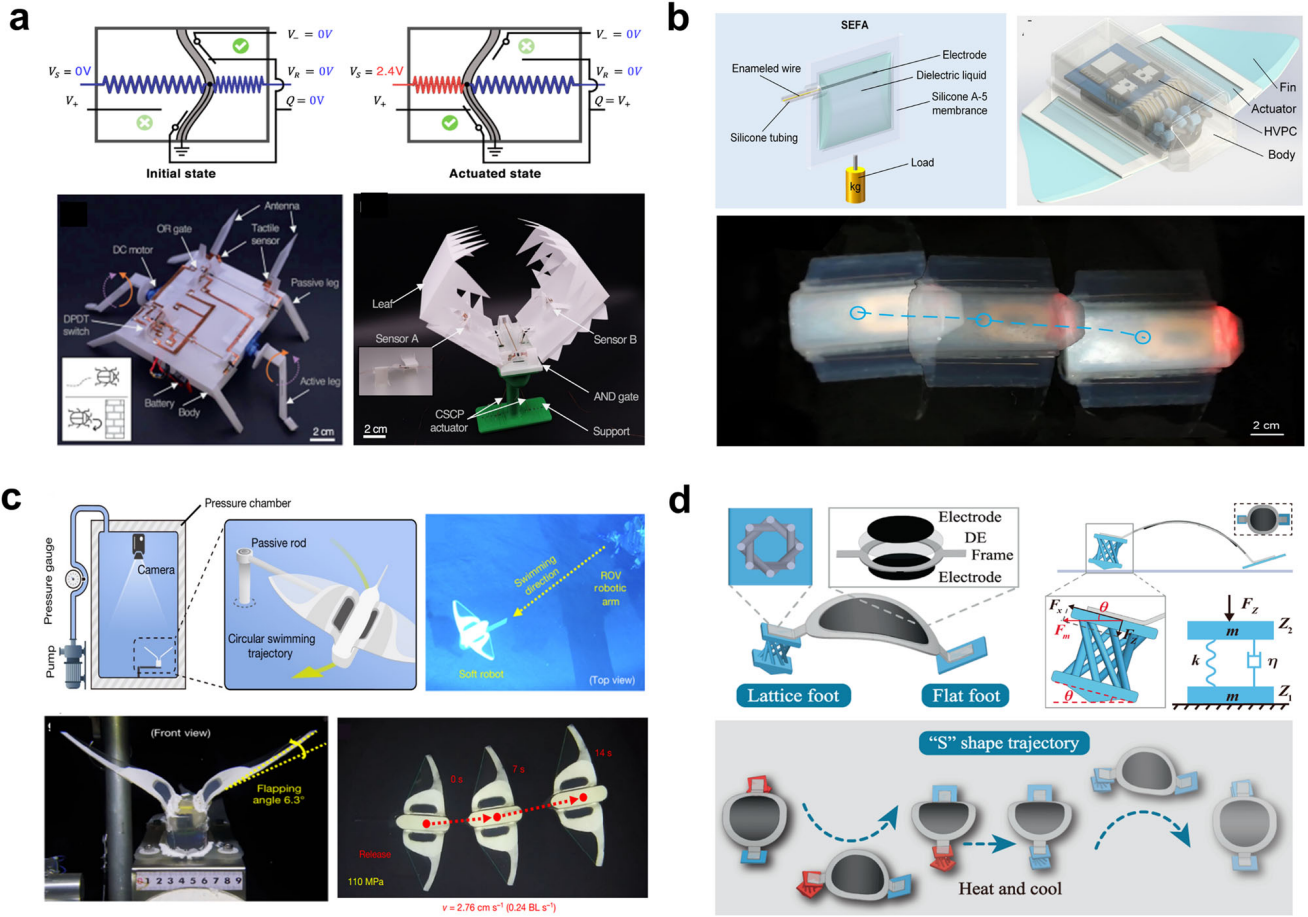
Untethered electrically-driven soft actuators. **a** Origami-based robot with autonomous functionality. Reproduced with permission under CC BY 4.0 license from ref. ^146^. **b** Untethered powered Untethered soft electrofluidic actuator that uses electrically responsive fluids. Reproduced with permission under CC BY-NC 4.0 license from ref. ^142^, copyright 2021, AAAS. **c** Bio-inspired snailfish-like untethered soft underwater robot. Reproduced with permission from ref. ^18^, copyright 2021, Springer Nature. **d** Dexterous electrically-driven soft robots with reconfigurable design. Reproduced with permission under CC BY 4.0 license from ref. ^154^.

The electrically-driven soft actuators are the type of actuators that operate with the electrical input. The use of dielectric elastomer actuators in Fig. 7 exemplifies this since these actuators produce mechanical deformation in the presence of voltage application. Electrically-driven soft actuators present significant benefits in untethered soft robotics, including versatile actuation mechanisms, mechanical flexibility, swift response times, and adaptability to a multitude of environments^95^. Various types of electrically-driven soft actuators have emerged, harnessing materials like liquid metals, shape memory alloys, conducting polymers, and hydrogels. These actuators offer versatile capabilities, with electronic signals facilitating precise control of their motion characteristics, and they can be seamlessly integrated with electronic devices and drivers, making them invaluable in fields like microfluidic systems^142,143^, manipulation of micro/microscale object^18,144,145^, and bio-inspired microrobots^146–148^. In one exemplary work, an origami-inspired approach integrated sensing, computing, and actuation directly into pliable and conductive materials, thus developing an autonomous robot with the capacity to interact dynamically with its environment (Fig. 7a)^146^. This integrated approach not only enhances the autonomy of the robots but also leverages the advantages of origami-based fabrication, offering multiple functionalities and a versatile platform for future innovations. In many cases, such actuators utilize electroactive polymers or dielectric elastomers that can generate substantial force output and potentially operate at higher frequencies^149^. Also, other researchers allowed the creation of a subgram, insect-sized robot capable of autonomously navigating and carrying payloads five times its weight using low-voltage stacked dielectric elastomer actuators (LVSDEAs) operating below 450 volts, showcasing the potential for resilient and fast untethered robots in soft robotics research^144^. Additionally, a representative example in Fig. 7b shows the development of self-contained soft electrofluidic actuators (SEFAs) that are fabricated based on basic techniques with widely accessible materials, demonstrating exceptional safety, reliability, controllability, durability, versatility, and swift response^142^. However, technical challenges remain in the high operating voltages due to the use of dielectric liquid along with the risk of dielectric breakdown if the actuator undergoes excessive strain^150,151^.

Addressing the need for environmental adaptability^152,153^, a group of engineers achieved a significant breakthrough with the design of an untethered and self-powered, electrically-driven soft robot fit for deep-sea exploration as illustrated in Fig. 7c^18^. This distinctive robot employs electrically-driven actuation to traverse deep-sea environments, safeguarding its integral electronics against extreme pressure by encapsulating them within a silicone matrix. Likewise, another research concentrated on enhancing soft robot mobility through a chiral-lattice design method, allowing immediate direction changes and complex motions controlled by voltage frequency (Fig. 7d)^154^. This approach marks a significant leap toward versatile, autonomous soft robots, capable of navigating complex environments and executing diverse tasks with remarkable agility and precision. Furthermore, the researchers developed a manta ray-inspired soft electronic fish using a dielectric elastomer actuator, with safety and stability enhanced by utilizing encapsulated hydrogel and surrounding tap water as an electric ground^148^.

In conclusion, electrically-driven actuators offer significant advantages, including quick response times and the potential for high-force output, making them well-suited for a wide range of applications. These actuators leverage principles like electromagnetic actuation and piezoelectric materials, allowing them to generate substantial mechanical forces. Their capability for high-force output is particularly valuable in scenarios where robust mechanical power is essential, such as robotics and automation. However, it is important to note that the exact force levels achievable with electrically driven actuators can vary depending on factors like their design, materials, and the specific application context. While they have the potential for high-force output, the actual force generated may need to be optimized to meet the unique demands of each application.

Furthermore, while electrically driven actuators are generally less sensitive to factors like temperature, light, and moisture when compared to some other types of soft actuators, they can exhibit sensitivity to factors like electrical interference, electromagnetic fields, and power supply stability. These considerations are crucial for ensuring their reliable performance in real-world applications. Additionally, extreme environmental conditions, such as exposure to moisture or high humidity, can impact the electrical components and safety of electrically driven actuators. Therefore, careful design and environmental management are essential to maximize their effectiveness and safety. In summary, electrically driven actuators have the potential to deliver high-force output, but the actual force levels may vary based on design and application. Their performance advantages must be balanced with considerations of environmental sensitivity and other application-specific factors to ensure their successful deployment in diverse environments and applications.

## Integration strategies of soft actuators into soft robotics

Based on a variety of soft actuators, as we discussed, to develop fully soft robots with untethered operation systems, a myriad of research has been devoted to integrating the various soft actuators with functional components such as sensors and powering sources^18,38,155^. For instance, as aforementioned, a group of researchers developed an untethered soft robotic fish by incorporating a printed circuit board and battery module^35^. This exemplifies a typical approach in which soft actuators are integrated into soft robotic systems alongside conventional functional components such as circuit boards, power sources, and microelectronics, which are rigid and bulky^34^. Nevertheless, this approach poses several challenges, including the potential for mechanical mismatch arising from the integration of rigid components into soft materials. Furthermore, there are concerns related to the potential scaling issues of soft robotic systems to accommodate a diverse array of functional components. These limitations necessitate further investigation and careful design considerations in order to effectively address their implications.

To address these challenges, extensive research has focused on developing soft sensors^156^, energy storage^157^, and energy harvesters^158^ to replace bulky components in recent decades, thereby establishing fully soft robots with untethered operation systems. Nevertheless, the critical issues still remain challenging, including the complex integration process and the delicate issue of mechanical mismatch. One viable solution is to achieve a unified soft robotic system by seamlessly integrating functional capabilities during the manufacturing stage^159–161^. For instance, Dong et al. provided seamlessly multilayered coaxial fibers that can self-sense and actuate as all-in-one artificial fibers^162^. Moreover, a smart humidity-responsive robot recently demonstrated remarkable intelligence in object manipulation and perception, exhibiting excellent stability across a broad range of relative humidity levels (10 ~ 75%)^163^. This robot autonomously generates distinct electrical signals corresponding to the type of objects that it interacts with. This functionality is accomplished through the triboelectric effect, initiated by contact between the objects and the gripper, which functions as a soft gripper. Consequently, the smart robot achieves simultaneous actuation and perception without the need for additional integrated components, advancing the development of lightweight and intelligent robotic systems.

Furthermore, for active control of systems, another research group devised a feedback control platform designed to maintain a target temperature without the need for a dedicated temperature sensor. This achievement is made possible by leveraging the temperature-dependent resistance of a nanowire heater, which is seamlessly integrated into the robotic systems, serving both as a heat source and an indirect parameter for temperature estimation^164^. These considerable endeavors have been directed toward reducing the complexity associated with robotic systems, thereby leading to the advancement of smart soft robotics.

Likewise, in the endeavor to achieve fully intelligent soft robotics with untethered operation systems, inherent challenges arise within the realm of sensing part. These issues encompass nonlinear output and hysteresis^165–168^, which not only complicate integration but also pose significant challenges in achieving proprioception among soft actuators. Moreover, the limitations associated with relying on a single sensor prevent complete self-sensing capabilities. To combat these challenges, significant efforts have been dedicated to leveraging machine-learning techniques as a promising solution^169^. For example, Shu et al. developed proprioceptive electronic skins enhanced by machine learning technology. These integrated systems endowed soft robots with outstanding capabilities to perceive and adapt to their surrounding environment, which implies their potential for practical, real-world applications^170^.

## Challenges and persepctive methods to overcome

The recent advances in soft actuators have bestowed the soft robots with fascinating functionalities as soft actuators are responsible for generating mechanical force, and especially the new type of untethered soft actuators have a promising potential to be applied in various fields such as autonomous robotics, smart haptic devices, and untethered medical robots. We believe that the future perspective of soft actuators and robots is clear: they should find the applications, to which the traditional robots can not be applied due to their rigid bulky structures. In the previous sections, we have thoroughly reviewed several types of untethered soft actuators that were published recently, and untethered soft actuators of different kinds have suitable applications of their own. For instance, it will be quite challenging to apply pneumatic and hydraulic soft actuators for drug delivery or invasive surgery in the form of a millimeter robot since pneumatic and hydraulic soft actuators require fluid chambers that take up a substantial volume. Instead, it is more suitable for pneumatic or hydraulic soft actuators to be applied to autonomous robotics^34,35,48,171^, haptic devices^49^, or assistance in medical procedures^172–176^ because they can generate a greater amount of force when compared to other untethered soft actuators. Especially for medical rehabilitation therapies, soft robotic exoskeletons or exosuits based on untethered pneumatic actuators can provide enough force to support and assist to individuals with mobility impairments or help with rehabilitation therapies, as opposed to other types of untethered actuators. For drug delivery or surgical applications, untethered magnet-driven soft actuators have desirable properties as their movements and configuration can be controlled accurately from outside by the magnetic field^177,178^.

Furthermore, as opposed to pneumatic and hydraulic soft actuators that demand artificial input, some of the external stimulus-driven actuators receive input from the natural surroundings, and such naturally occurring inputs have profound implications for the soft actuators as they can be easily associated with physical intelligence. Physical intelligence is another field of study that receives much attention from soft robotics since it does not incorporate artificial intelligence or human input for its movements. Physical intelligence can be defined as the physical encoding of an agent’s sensing, actuation, control, memory, logic, computing, adaptability, learning, and decision-making capabilities^179^, so it requires a collective integration of interdisciplinary studies to encode physical intelligence into the thoroughly designed soft robot since it requires the careful selection of materials and structural design for both sensing and soft actuator to enhance the maximized functionalities of the soft robot. As discussed previously, several types of robots that are driven by naturally occurring external stimuli such as heat^180,181^, light^182–185^, humidity^186–189^, or even multiple stimuli^190^, fall into this category. Physical intelligence offers novel functionalities for untethered external stimulus-driven soft actuators because soft actuators can produce cyclic movements even in the absence of artificial inputs or human intervention, making them highly autonomous. Furthermore, soft robots with physical intelligence are capable of sensing and actuating with the single device structure, suggesting that they do not need to incorporate additional onboard circuitry for embedded artificial intelligence and sensors that might interfere with the general movement of the soft robots.

Despite the fascinating properties and feasible applications of untethered soft actuators, there exist several challenges that soft actuators need to address. The most significant challenge of soft actuators would arise from a seamless integration of core components into a functional untethered soft robot. For instance, even if the arbitrary soft actuator is capable of generating a considerable amount of mechanical force, the ideal type of soft robot consists of various sensors, embedded artificial intelligence, and a power source, which usually take the physical form of the printed circuit board (PCB) and battery. Therefore, the incorporation of circuitry and battery components might add unwanted weight and volume that might degrade and even conflict with the performance of the soft actuator.

Along with the added components that might impair the actuating efficiency, soft actuators often lack built-in sensing capabilities, making it challenging to obtain real-time feedback on their deformation and force output. Despite remarkable technological advances in soft sensors, soft sensors still lack reliability and accuracy when compared to rigid commercial sensors, and thus integrating sensors into soft actuators or developing external sensing mechanisms to provide feedback for closed-loop control can be a complex task. Also, the lack of accuracy and reliability of soft sensors can complicate the robotic control and feedback mechanism since soft actuators often exhibit non-linear and complex behavior^191^, making their control and precise manipulation challenging. In this regard, developing control strategies and feedback mechanisms that can accurately regulate the movement and deformation of soft actuators is crucial for achieving desired robot behaviors.

Another challenge the soft actuators need to overcome is modulus mismatching with rigid materials. Since Young’s modulus of soft robots matches that of the human skin, the human-machine interfaces serve as one of the most promising applications of soft robots. The soft and flexible nature of soft robots makes them more comfortable and safer to interact with humans. Nevertheless, there exists a demand in which both soft and traditional robotics are employed in a hybrid form. In this case, modulus mismatching can become a critical factor that affects the general performance since it can affect force transmission and control. Modulus mismatching can influence the transmission of forces from the actuator to the end effector of the soft robot or the object it interacts with. Thus, optimization of the stiffness of the actuator relative to other components should be carried out carefully such that the robot can transmit forces efficiently and precisely, enabling accurate manipulation or locomotion.

Besides, the durability and reliability due to soft and compliant material makeup can cause considerable issues for soft actuators. Soft actuators, especially those made of elastomeric materials, are vulnerable to fatigue and wear over time, and such a change in the mechanical property due to repeated use can affect the performance of soft actuators. Ensuring the durability and long lifespan of soft actuators is important for practical applications, as they need to withstand repetitive movements and deformations without significant degradation in performance.

## Outlook and potential oppurtunities

Despite the incredible advances in soft actuators and their applications in robotics as discussed in this article, the research scope and the applications of the soft actuators still pertain to the academic fields. Rather, for soft actuators to find their true meaning and significance, they need to be utilized in the industry or deployed in the real-life field to deliver their purposes. As discussed throughout this article, soft actuators possess the physical properties and other pertinent features that traditional rigid actuators cannot emulate, so certain real-life applications surely exist that only soft actuators can deliver. For instance, for wearable exoskeletons or movement-assistive devices, the use of soft actuators can reduce the chance of injury or damage due to their soft and flexible nature. To a certain extent, soft actuators show definite advantages when interacting with human interaction or delicate and mechanically fragile objects. Furthermore, if the entire robot is comprised of soft materials, it would allow the soft robot to be deployed to travel through the unstructured and complex environment or even to a deep sea where pressure is extremely elevated – a task that the traditional rigid robots cannot deliver. Nevertheless, the following issues should be fully addressed for the industry commercialization of soft actuators in the overall perspective of soft robotics.

### Reducing fabrication cost of soft robotic actuators

Apart from market readiness and limited awareness of soft robotics, the authors believe that the hurdle mainly arises from fabrication costs. Currently, the fabrication of soft actuators and the entire integration into soft robotic systems are conducted on the laboratory scale. Thus, the cost to manufacture the soft actuator and assemble core components to make a soft robot remains considerably higher than that to manufacture a rigid robot. Nevertheless, the promising realm of 4D printing, an innovative fabrication technique, presents unique advantages and features that hold the potential to mitigate these challenges^192^. Diverging from conventional 3D printing, 4D printing integrates materials capable of dynamic transformations in response to external stimuli, such as temperature, humidity, or environmental cues. This exceptional capability empowers the creation of intricate, self-assembling structures and sophisticated soft actuators endowed with programmable behaviors^193^. Despite the emergence of scalable fabrication approaches in the realm of soft robotics, including the compelling avenue of 4D printing, these methodologies have not yet achieved widespread acceptance. This lag in adoption primarily stems from the absence of standardized practices. Since the concept of soft robotics was newly coined in 2012^194^, the field lacks standardization in terms of materials, design, and control systems, which makes it difficult for the industry to adapt soft robots into their workflow. Moreover, the fabrication recipes. Therefore, the collective efforts from academia and industry are required to reduce and further introduce cost-effective soft robots.

### Manufacturing reproducibility and consistent force output

Another issue in the manufacturing side of soft actuators and system integration originates from manufacturing reproducibility and consistent force output. For widespread use and industrial commercialization of soft robots, large-scale manufacturing reproducibility must be guaranteed since the physical properties of the soft materials might be altered even with a minute change in the environmental condition. For instance, humidity and manufacturing temperature can affect the hydrogel-based soft actuators as these factors contribute to variations in the moisture contents inside the hydrogels. Also, the chemical composition of chemical precursors to make finalized materials might vary from batch to batch when it comes to large-scale processing, affecting manufacturing reproducibility in general. The deviation of manufacturing reproducibility can result in inconsistent force/torque output in soft actuators, and this can affect the overall performance and reliability of soft robots. Fortunately, the significant advances in automated technologies that are controlled at a highly precise level should be able to address the manufacturing reproducibility of soft robots in the near future.

### Consistent and precise force output

One of the main future works that soft actuators have to attain is to generate consistent and precise force output in the presence of the programmed input. Although structural design and material selection can contribute to achieving precise force control to a certain extent, soft actuators cannot produce as precise and consistent force output as traditional rigid actuators due to their inherently compliant material composition. As discussed earlier, this can pose a serious problem since it can eventually lead to reliability issues despite all the favorable properties of the soft actuators. Nevertheless, there are several means to improve the consistency and preciseness of the soft actuators. For instance, due to the rapid developments of soft sensors in a variety of types, the soft sensors can be integrated with the soft actuators to establish the sensory-haptic control feedback such that the force output can be adjusted in real-time. Another way to address this issue is to incorporate machine learning into the soft robot for precise force generation after training. In this regard, although soft actuators alone possess comparative weaknesses over rigid actuators in precise force generation, the system integration with the control algorithm can compensate for the core limitation of the soft actuators.

### Material durability

Due to the compliant and deformable material nature of soft actuators, material durability, including mechanical wear and tear over time as a result of fatigue stress with the industrial setting of high-frequency usage, remains the essential issue to be addressed for the widespread usage of soft actuators in the imminent future because it can definitely affect the performance of the soft actuators. For instance, it can affect the actuation range, precision, force output, response time, and even failure mode. Even though the development of soft yet robust materials can be a direct and definite answer to the fatigue durability issue, roboticists should also invest their efforts to optimize the design of the actuator to minimize the stress concentration and reduce the strain on critical regions.

## Discussion

Addressing the challenges of soft actuators requires interdisciplinary research efforts combining materials science, robotics, control engineering, and manufacturing technologies. In other words, it implies that there still exist plenty of opportunities for soft roboticists to contribute to developing soft actuators further to their practical commercialization. Continued advancements in these areas will contribute to the development of more capable and robust soft actuators, enabling the broader adoption of soft robots in various applications.
